# For and against tumor microenvironment: Nanoparticle-based strategies for active cancer therapy

**DOI:** 10.1016/j.mtbio.2025.101626

**Published:** 2025-03-01

**Authors:** Soroush Karimi, Roksana Bakhshali, Soheil Bolandi, Zahra Zahed, Seyedeh Sahar Mojtaba Zadeh, Masoumeh Kaveh Zenjanab, Rana Jahanban Esfahlan

**Affiliations:** a Nano Drug Delivery Research Center, Health Technology Institute, Kermanshah University of Medical Sciences, Kermanshah, Iran; b Omid Cancer Center, Ahvaz, Iran; c Shiraz University of Medical Sciences, Shiraz, Iran; d Department of Medical Sciences, Ardabil University of Medical Sciences, Ardabil, Iran; e Department of Pharmaceutics, Faculty of Pharmacy, Tehran University of Medical, Sciences, Tehran, Iran; f Department of Medical Biotechnology, Faculty of Advanced Medical Sciences, Tabriz University of Medical Sciences, Tabriz, Iran

**Keywords:** Tumor microenvironment modulation, Engineered nanoparticle, Cancer therapy

## Abstract

Cancer treatment is challenged by the tumor microenvironment (TME), which promotes drug resistance and cancer cell growth. This review offers a comprehensive and innovative perspective on how nanomedicine can modify the TME to enhance therapy. Strategies include using nanoparticles to improve oxygenation, adjust acidity, and alter the extracellular matrix, making treatments more effective. Additionally, nanoparticles can enhance immune responses by activating immune cells and reducing suppression within tumors. By integrating these approaches with existing therapies, such as chemotherapy and radiotherapy, nanoparticles show promise in overcoming traditional treatment barriers. The review discusses how changes in the TME can enhance the effectiveness of nanomedicine itself, creating a reciprocal relationship that boosts overall efficacy. We also highlight novel strategies aimed at exploiting and overcoming the TME, leveraging nanoparticle-based approaches for targeted cancer therapy through precise TME modulation.

## Graphical abstract

**Figure:**
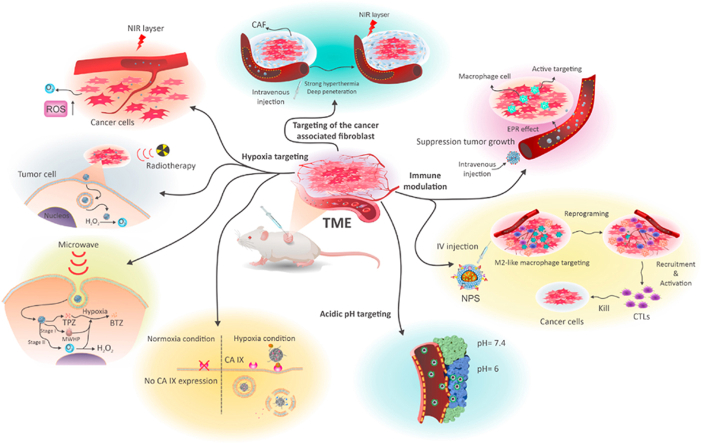

## 1 Introduction

Cancer presents a significant challenge, with its intricate and dynamic nature often evading conventional treatment approaches [1]. The tumor microenvironment (TME), characterized by its diverse cellular components, extracellular matrix (ECM), and complex signaling networks, significantly impacts tumor growth, metastasis potential, and resistance to standard therapies [2,3]. Key features of the TME, such as hypoxia, acidic pH, and immune suppression, not only promote tumor progression but also offer unique opportunities for targeted interventions.

Nanotechnology is emerging as a promising frontier, offering new approaches to modulate the TME and overcome obstacles in the way of successful cancer treatment [4]. NPs (NPs), with their numerous applications, can specifically interact with and control important TME components, thereby enhancing the effectiveness of cancer therapy [5,6].

A crucial strategy for adjusting the TME is to address the problem of tumor hypoxia. Hypoxia, a characteristic of solid tumors, limits the effectiveness of treatments, which is a major obstacle to effective therapy [7,8]. By enhancing local oxygen levels—either through oxygen-generating mechanisms, improved blood flow, or reduced consumption—these NPs optimize the conditions for effective chemotherapy and other treatments. Targeting hypoxia-inducible factors with specific nanoparticle formulations represents a strategic advancement, allowing for precise and tailored therapeutic interventions that improve outcomes [[9], [10], [11]]. Tumor hypoxia-targeted drugs represent another avenue to selectively act on the hypoxic regions and improve overall cancer treatment efficacy.

In addition to treating hypoxia, NPs can take advantage of the TME's acidic pH. Drug delivery to cancer cells can be enhanced while limiting off-target effects by utilizing pH-responsive mechanisms in nanoparticle-based platforms, which enable the selective release of therapeutic payloads in the acidic TME [12,13]. Certain NP systems have the ability to counteract the excess acidity in the TME, which may hinder tumor growth while providing a better setting for cancer therapy [13].

NPs can modulate immune elements by reprogramming TME-associated macrophages and strategically delivering immunostimulatory agents, thus enhancing anti-tumor immunity. Moreover, advances in nanotechnology enable precise targeting and manipulation of interactions within the TME, offering new pathways in oncological treatments [9,14].

Additionally, targeting interactions between cancer cells, cancer-associated fibroblasts (CAFs), and the ECM with NPs can disrupt tumor-supporting frameworks and enhance therapeutic susceptibility [9,15].

This paper presents a comprehensive and innovative perspective on leveraging nanotechnology to reshape the TME and enhance cancer therapy outcomes. By integrating cutting-edge nanoparticle-based strategies with a deep understanding of TME complexities, the scientists aim to set the stage for a transformative approach to efficient and targeted cancer treatment. The insights offered pave the way for further exploration and development of nanoparticle-mediated interventions to address the challenges posed by the intricate dynamics of the TME in cancer progression and treatment.

## 2 TME properties and roles in tumor growth

TME is a sophisticated and dynamic system that has a substantial influence on the growth of cancer and the extent to which it is resistant to treatment. There are many different components that make up this system, such as the ECM, blood vessels, immune cells, fibroblasts, and other stromal elements. These components all collaborate with cancer cells to create a specific environment that is favorable to tumour growth and the spread of metastases. One of the defining aspects of the TME is its complexity and heterogeneity, which have a significant impact on the behaviour and therapeutic responses of cancer cells [16,17]. This intricacy enables the TME to boost cancer cell proliferation, enhance survival mechanisms, facilitate invasion of adjacent tissues, and enable immune evasion methods, according to preliminary investigations. For example, the flexible nature of the not only helps to provide structural support for tumour cells, but it also helps to regulate important signalling pathways that are associated with cell growth and migration. The abnormal blood vessels that are typical of the TME, which are often very disorganized and irregular, affect the flow of oxygen and nutrients, which in turn affects how well cancer cells can adapt and how well they can resist treatment [18,19]. As a result of hypoxia, which is frequent in the TME, the oxygen supply is limited, which forces cancer cells to adapt by switching to anaerobic metabolism. This allows the cancer cells to survive and enables further growth of the tumour. Additionally, it stimulates angiogenesis, which helps tumors acquire a greater blood supply. At the same time, the acidic pH that develops as a consequence of increased hydrogen ion generation has an effect on the remodeling of the ECM. It makes invasion easier by destroying barriers and helps immune evasion by reducing the function of immune cells [20,21].

## 3 NPs in targeting the TME

NPs have emerged as a promising tool to overcome challenges presented by the TME. The Enhanced Permeability and Retention (EPR) effect makes it easy for NPs to gather in the TME [22]. They can be engineered with targeting ligands that recognize specific receptors on cancer cells or stromal components, ensuring precise delivery of therapeutic agents to tumour sites while minimizing systemic toxicity [23]. Because they can get through the thick extracellular matrix and abnormal vasculature, NPs are especially effective at treating acidic and hypoxic areas that don't respond well to other treatments [24]. NPs' multifunctionality allows them to carry various agents, such as chemotherapeutics, gene therapies, or imaging agents, which facilitate simultaneous treatment and diagnostic applications. This enables real-time monitoring of therapy responses. By adding immunomodulatory agents, NPs can change the immunosuppressive environment in the TME, which boosts the immune system's ability to fight tumors [25]. They are also designed to respond to specific TME stimuli like pH and temperature, enabling controlled drug release that maximizes therapeutic efficacy while minimizing harm to healthy tissues. Also being worked on are nanotherapeutic strategies that are designed to target specific features of the TME and get into complex tumor structures [25,26].

## 4 Hypoxia-targeting NPs to enhance therapy

Hypoxia, a common feature in TMEs, complicates treatment efforts by limiting oxygen availability and promoting malignancy. Hypoxia in tumors promotes treatment resistance, tumor activity, and immune evasion, making it a serious obstacle to the development of successful cancer therapies [27,28]. NPs engineered to target hypoxic regions can enhance drug delivery and therapeutic outcomes. For example, Wang et al. created NPs that improve the results of cancer treatment by increasing the amount of oxygen that gets to tumors [29]. Similarly, Thambi et al. reported self-assembled polymeric NPs that release therapeutic agents under hypoxic conditions, offering a targeted approach for drug delivery in tumors [30]. These methods use the low-oxygen environment to deliver drugs more precisely. They do this by getting around biological barriers and using hypoxia-responsive drug release systems and other methods [31].

Table 1 lists the NPs that are effective in mitigating the effects of hypoxia.

**Table 1:** Exploiting and Overcoming the TME by applying NPs in hypoxia condition.

Strategies based on NPs	Techniques based on NPs	NPs
Enhancing Oxygen in Hypoxia Condition	Oxygen Delivery	Perfluorocarbon NPs [32], Polydopamine-NP-stabilized oxygen microcapsules [33]
	Generating oxygen in hypoxic tumors	Manganese dioxide [34], polydopamine@manganese dioxide [35], carbon-dot-doped C3N4 nanocomposite [34,36], CaO2 NP formulation coated with a pH-sensitive polymer [37], multifunctional chlorine e6 (Ce6) loaded MnO2 NPs with surface PEG modification (Ce6@MnO2-PEG) [38].
	Alleviating tumor hypoxia by enhancing blood flow and reducing oxygen consumption	gold nanoshells [39], Liposomes [40]
Tumor hypoxia-targeted drugs	Oxygen-dependent treatment modalities	microwave-sensitized nanomaterial [41], silica-coated upconversion [42], micelles [43]
Targeting Hypoxia-Activated Receptors	Targeting activate specific receptors	porous silicon [44], nanomicelles [45]

### 4.1 Enhancing oxygen in hypoxia condition

TME's hypoxic circumstances severely restrict the effectiveness of cancer treatments. By increasing blood flow and lowering oxygen consumption, or by directly supplying oxygen, NPs can be engineered to raise oxygen levels in the TME [15]. This leads to better therapeutic outcomes because it makes tumors more susceptible to the cytotoxic effects of different treatment regimens. Nanoparticle-based strategies can increase tumor cells' vulnerability to the cytotoxic mechanisms of cancer treatments by addressing the underlying hypoxia [46]. This strategy has the ability to realize the potential of cancer treatment and overcome a significant obstacle presented by the hypoxic TME.

#### 4.1.1 Oxygen delivery

**Fig. 1:**
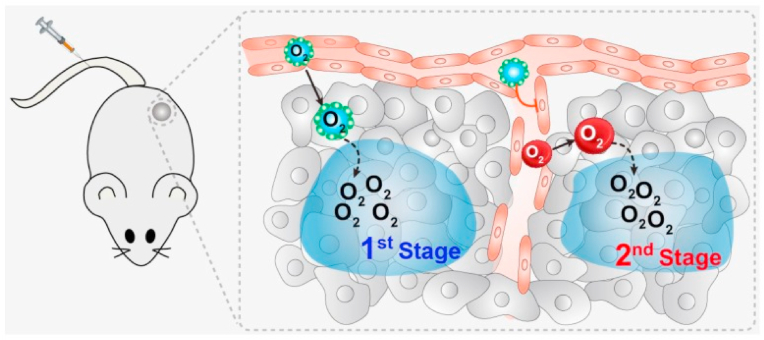
Two-stage nanoparticle-based oxygen delivery system -stage nanoparticle-based oxygen delivery system to address the hypoxic tumor microenvironment. PFTBA@HSA NPs first accumulate in the cancer site by the EPR effect, releasing physically bound oxygen. Secondary, they disrupt tumor vasculature, enabling increased red blood cell infiltration and secondary oxygen supply to the hypoxic regions [49].

One of the key ways that NPs overcome hypoxic situation is by supplying oxygen directly to the TME. NPs primarily increase local oxygenation by delivering and releasing oxygen in hypoxic areas. This technique reduces the oxygen deficiency that increase the effectiveness of numerous cancer therapies [47]. Furthermore, NPs carried with catalase can degrade hydrogen peroxide in the tumor into water and oxygen, boosting local oxygen levels and possibly changing hypoxia-induced resistance to therapies such as radiotherapy and chemotherapy [48]. Notable studies include Zaigang Zhou et al.'s development of a two-stage PFC NP-based oxygen delivery system, which showed improved radiotherapy efficacy in hypoxic tumors. This method involves initial oxygen release, followed by promoting red blood cell infiltration for sustained oxygen delivery. Similarly, bismuth selenide NPs loaded with PFC, when irradiated with near-infrared light, release oxygen to significantly reduce hypoxia and enhance radiotherapy outcomes via increased ROS production (Fig. 1) [49].

In another study, investigators developed hollow bismuth selenide (Bi_2_Se_3_) NPs and loaded them with PFC. When near-infrared (NIR) light hits these PFC-loaded Bi_2_Se_0_ NPs, they give off oxygen because Bi_2_Se_0_ is photothermal. In vivo experiments using mice bearing tumors demonstrated that the NPs accumulated in tumors and, upon NIR irradiation, released oxygen, significantly decreasing hypoxia and augmenting the effectiveness of radiotherapy by elevating ROS production [50]. Cheng et al. developed PFC-based NPs encapsulated in a lipid shell, which form stable oxygen carriers. They injected these NPs into tumor-bearing mice, and the enhanced EPR effect caused them to accumulate in the TME. The oxygen-loaded PFC NPs boosted the levels of oxygen within the tumors, reducing hypoxia and improving the efficacy of PDT, leading to more effective tumor reduction [51]. Researchers designed a multifunctional nanoplatform (ZDZP@PP) that encapsulates the photosensitizer protoporphyrin IX and the chemotherapeutic drug doxorubicin. The ZIF-67 core of the platform acts as a hydrogen peroxide catalyst, while the ZIF-8 shell has pH-responsive properties. By generating oxygen from the breakdown of hydrogen peroxide, it alleviates tumor hypoxia and increases the effectiveness of combined chemo-photodynamic therapy for better cancer treatment when compared to separate therapies in vitro and in vivo [52]. To improve the effectiveness of PDT and address hypoxia in the TME, researchers have developed a unique NPs technology. The platform creates a continuous oxygen-evolving system by combining MnFe3O4 NPs anchored onto mesoporous silica NPs (MSNs). To get around the lack of oxygen, MnFe2O4 NPs speed up the breakdown of H2O2 in the TME, which creates oxygen right there. MSNs carry photosensitizers, which are essential for PDT. By addressing a major issue brought on by the hypoxic TME, this dual-functionality method demonstrated considerable tumor growth inhibition in vivo, indicating the potential of this nanoparticle system to improve PDT outcomes [53].

#### 4.1.2 Generating oxygen in hypoxic tumors by applied NPs

**Fig. 2:**
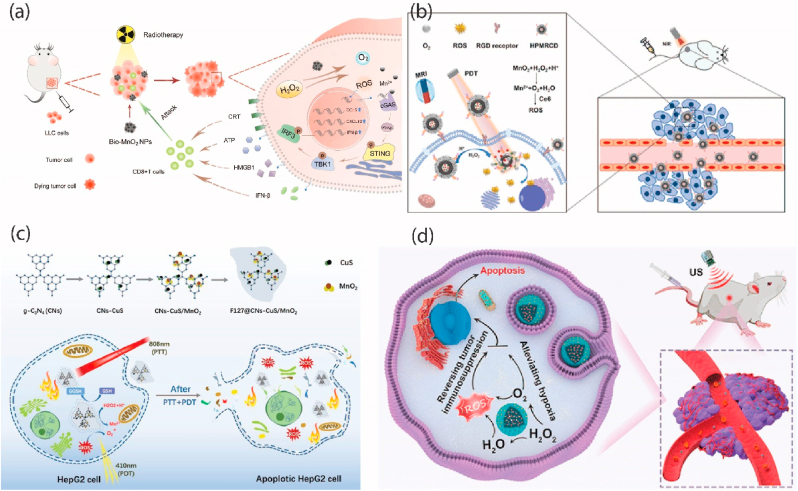
(a) Biomineralized Manganese Oxide NPs Synergistically Relieve Cancer Hypoxia and Activate Immune response with Radiotherapy in Non-Small Cell Lung Cancer. Copyright by MDPI 2022 [56]. (b) Biodegradable Hollow Polydopamine@manganese Dioxide as an Oxygen Self-Supplied NPs for enhancing Chemo-photodynamic Target tumor. Copyright 2021 American Chemical Society [35] (c) The multifunctional nanoplatform, based on g-C3N4, is loaded with MnO2 and CuS NPs for oxygen self-generation, photodynamic, and photothermal synergistic therapy. Copyright by Elsevier 2022 [57] (d) A cascade nanozyme that amplifies sonodynamic therapeutic effects by comodulating hypoxia and immunosuppression against cancer has been developed. Copyright by American Chemical Society 2021 [58].

To overcome the limitations imposed by hypoxia, researchers have developed NPs that can either generate oxygen on-site or deliver it directly to tumor locations. MnO_2_ has shown promise as an agent for oxygen delivery to hypoxic tumors. It reacts with the elevated levels of hydrogen peroxide (H_2_O_2_) commonly found in the TME, forming manganese ions (Mn^2+^) and O_2_ [34,54]. This reaction effectively elevates local oxygen levels within the acidic environment of tumors, where the pH is approximately 6.0, thereby enhancing the interaction between NPs and the TME [34,55]. Manganese dioxide (MnO_2_) has emerged as a promising agent for oxygen production. In hypoxic conditions, MnO_2_ reacts with the elevated levels of hydrogen peroxide (H_2_O_2_) typical in TMEs, generating oxygen while releasing manganese ions (Mn^2+^). This reaction effectively occurs in the acidic environment of tumors (pH ∼6.0). Xinyu Liu et al. demonstrated that biomineralized MnO_2_ NPs can not only alleviate hypoxia in non-small cell lung cancer (NSCLC) but also enhance immune response and radiation therapy efficacy by inducing reactive ROS and activating immune pathways (Fig. 2 (a)) [56]. Wang et al. have designed biodegradable hollow polydopamine@manganese dioxide (PDA@MnO_2_) NPs as an oxygen self-supplied nanoplatform to enhance chemo-photodynamic cancer therapy. These NPs address tumor hypoxia by generating oxygen in response to elevated H_2_O_2_ levels in the TME. The hollow structure allows for efficient loading of chemotherapeutic agents (DOX), improving the efficacy of both PDT and chemotherapy (Fig. 2(b)) [35]. In another study, researchers designed a multifunctional platform, F127@CNS-CuS/MnO2, incorporating MnO2 NPs, g-C3N4, and CuS NPs. This comprehensive system tackled the challenges of single-mode cancer therapies by generating oxygen to mitigate tumor hypoxia, responding to elevated glutathione levels in cancer cells, and potentiating photothermal therapy (PTT) and photodynamic therapy (PDT) synergistically for enhanced therapeutic outcome (Fig. 2 (c)) [57]. Enhanced PDT in hypoxic tumors has been achieved using carbon-dot-doped C3N4 nanocomposite (PCCN), a carbon-dot-doped C3N4 nanocomposite. PCCN facilitates oxygen generation through photo driven water splitting, addressing hypoxia-induced PDT resistance and improving treatment outcomes [36]. Tao et al. demonstrated that gold and carbon dot-modified TiO_2_ NPs can reverse immunosuppression while generating ROS and oxygen to enhance sonodynamic therapy (SDT) effectiveness in cancer treatment (Fig. 2(e)) [58].

Zhang et al. designed a biomimetic nanoplatform encapsulating catalase and photosensitizers within a porous framework. This system autonomously generates oxygen, enhancing targeted PDT by evading immune detection and specifically accumulating in tumors [59]. Research by Tong Fan et al. highlighted a dual-drug NP approach that incorporates verteporfin and atovaquone to reduce mitochondrial oxygen consumption. This increases intracellular oxygen levels, significantly amplifying the antitumor effects of PDT [60].

#### 4.1.3 Strategies to alleviate tumor hypoxia

Hyperthermia increases the local temperature of tumors, thereby enhancing blood flow and consequently improving tumor oxygenation. This concept, demonstrated by the use of gold nanoshells in the perivascular space, shows significant improvement in radiation therapy outcomes. By synergistically combining antihypoxic and vascular disrupting therapies, this strategy can be integrated with conventional cancer treatments for enhanced effectiveness [61,62]. To improve treatment outcomes, researchers have developed various radioisotope-labeled, NIR-absorbing NPs to integrate PTT and radioisotope therapy. Radiotherapy comprises external methods such as X-rays and internal radioisotope therapy. Combining PTT and internal radioisotope therapy enhances anti-tumor efficacy [15,63]. Yu Chao et al. developed tungsten disulfide (WS) nanoflakes modified with polyethylene glycol (PEG), which can be labeled with radioisotope 188Re for enhanced radioimmunotherapy (RIT). Tungsten serves as a radiosensitizer, absorbing radiation and thus enhancing RIT efficacy. The WS_2_-PEG nanoflakes' strong NIR absorbance enables PTT, alleviating tumor hypoxia and overcoming radioresistance. The multifunctional 188Re-WS_2_-PEG system exhibited significant tumor ablation in mouse models through NIR-enhanced, self-sensitized cancer treatmen [63]. In addition to increasing oxygen supply, reducing oxygen consumption can also alleviate tumor hypoxia. R. Ansiaux et al. discovered that vandetanib, an inhibitor of VEGFR-dependent angiogenesis, effectively decreases the oxygen consumption rate of tumor cells, thereby enhancing tumor oxygenation and gradually improving the efficacy of radiotherapy [64].

### 4.2 Tumor hypoxia-targeted drugs

**Fig. 3:**
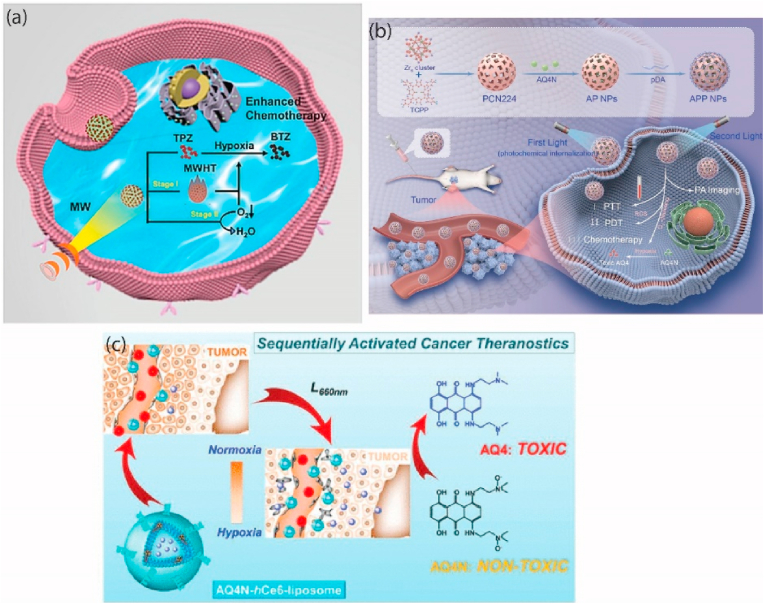
(a) A two-stage exacerbated hypoxia nanoengineering strategy induced an amplified activation of tirapazamine for microwave hyperthermia-chemotherapy of breast cancer [41]. Copyright (2024) Elsevier (b) The study focuses on photochemically-driven, highly efficient intracellular delivery and light/hypoxia-programmable triggered cancer photo-chemotherapy [43]. Copyright (2023) Springer Nature (c) Theranostic Liposomes with Hypoxia-Activated prodrug to effectively destruct hypoxic tumors post-photodynamic therapy [71]. Copyright (2016) American Chemical Society.

The recent focus has been on utilizing a combination approach in cancer therapy, which involves the use of chemotherapeutic drugs that specifically target and exhibit toxicity toward hypoxic cells, coupled with oxygen-dependent treatment modalities [65,66]. The combination of hypoxia-specific chemotherapy drugs and oxygen-dependent therapies has gotten a lot of attention because it has led to such amazing benefits for patients. The synergistic effects observed have been particularly noteworthy, showcasing the potential of this combinatorial strategy in enhancing the efficacy of cancer treatment regimens [67]. Tirapazamine (TPZ) is a noteworthy prodrug activated under hypoxic conditions, which specifically targets hypoxic tumor cells. This makes TPZ a valuable adjunct to radiotherapy, which primarily affects oxygenated cells. By attacking both hypoxic and oxygenated tumor cells, TPZ enhances overall treatment efficacy [68,69]. An innovative approach involving TPZ is the two-stage strategy using microwave-sensitized nanomaterial (GEMT). GEMT NPs, composed of GdEuMOF and TPZ, induce hypoxia by creating localized circulatory disturbance and passive hypoxia initially through microwave (MW) sensitization. Subsequently, these NPs exacerbate hypoxia by actively depleting residual oxygen. This dual-stage hypoxia amplification significantly potentiates TPZ activation, improving the outcomes of microwave hyperthermia and chemotherapy in breast cancer treatment Fig. 3 (a) [41]. Recent studies have developed a redox/pH-sensitive micelle system (PDM) conjugated with a naphthalimide-based prodrug (PNA) via disulfide linkage. These PDM micelles encapsulate the hypoxia-activated prodrug, banoxantrone (AQ4N), offering a stable drug delivery system that releases more drugs under acidic or redox conditions. The PNA prodrug also offers real-time monitoring capabilities due to its fluorescent properties, significantly enhancing hypoxia-targeted therapy efficiency. This innovative approach improves cancer combination therapies by integrating PNA chemotherapy with hypoxia-activated AQ4N therapy [70]. To improve the efficacy of cancer treatment, Wei Zhang and his colleagues investigated the development of a stimuli-responsive nanoplatform (APP NPs) that integrates photoacoustic (PA) imaging, phototherapeutic activities, and hypoxia-activated chemotherapeutic qualities. The chemotherapeutic medication AQ4N was loaded into the PCN-224 structure during the nanoplatform's development, and the dopamine monomer was hybridized there as well. The APP NPs efficiently induce cellular internalization, produce strong photothermal and photodynamic effects, and activate the hypoxia-targeted AQ4N with sequential light irradiation Fig. 3 (b) [43]. Liangzhu Feng et al. demonstrated a versatile liposome platform containing AQ4N and the photosensitizer hCe6, which is tagged with 64Cu for multimodal imaging. These liposomes achieve efficient tumor targeting, and upon light activation, they induce severe tumor hypoxia, which activates AQ4N. This results in a synergistic application of photodynamic therapy and hypoxia-activated chemotherapy, significantly enhancing cancer treatment outcomes Fig. 3 (c) [71].

Two drugs, evofosfamide (TH-302) and afatinib (AFT), are being studied to see how well they work together to treat nasopharyngeal carcinoma. Researchers designed folate-targeted mesoporous silica NPs to deliver the two drugs together. These particles worked better against cancer than the drugs alone [72].

### 4.3 Targeting hypoxia-activated receptors with NPs

**Fig. 4:**
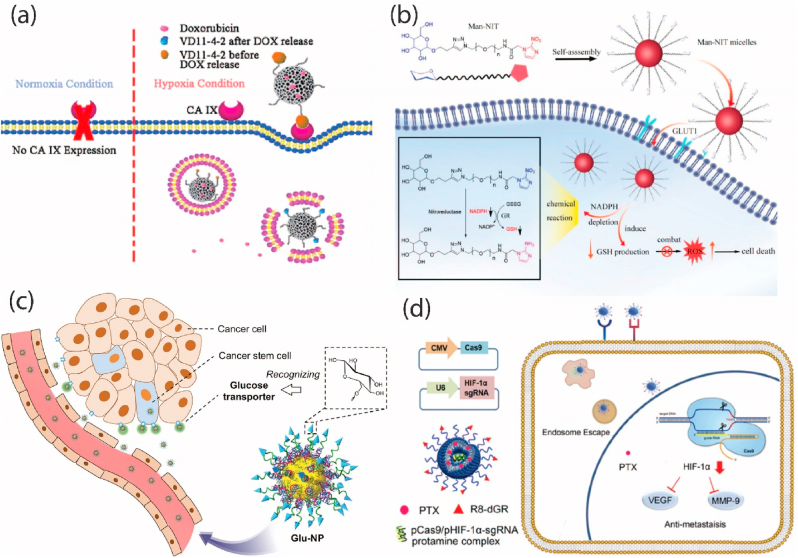
(a) An adaptable carbonic anhydrase IX targeting ligand-functionalized porous silicon nanoplatform for cancer therapy and imaging with low oxygen levels [44]. Copyright (2017) American Chemical Society (b) The use of nanomicelles for GLUT1-targeting hepatocellular carcinoma therapy relies on NADPH depletion [45]. Copyright (2023) Taylor & Francis (c) Glucose-linked sub-50-nm unimer polyion complex-assembled gold nanoparticles for targeted siRNA delivery to glucose transporter 1-overexpressing breast cancer stem-like cells [76]. Copyright (2019) Elsevier (d) The metastasis of pancreatic cancer was stopped by targeting the tumor with the CRISPR/Cas9 system and turning off hypoxia-inducible factor-1 alpha [77]. Copyright (2019) Elsevier.

The TME's lower oxygen concentrations have the ability to activate specific receptors, including the carbonic anhydrase IX (CAIX), glucose transporter 1 (GLUT1), and hypoxia-inducible factor (HIF) [73]. By avoiding hypoxia-related barriers and effectively penetrating cancer cells, NPs with this active targeting approach can improve medication delivery and therapeutic efficacy. Janoniene et al. developed a target-specific nanosystem using porous silicon (PSi) particles loaded with VD11-4-2, a novel CAIX inhibitor. This system exhibits a high affinity for hypoxic human breast adenocarcinoma (MCF-7) cells owing to CAIX's heightened expression under hypoxic conditions. The VD-PSi complex releases doxorubicin (DOX) under varying pH conditions, facilitating drug delivery and enhancing drug-resistant cancer cell death. The fluorescence resonance energy transfer effect allows for intracellular monitoring of DOX release (Fig. 4 (a)) [44]. Innovative nanomicelles, Mannose-polyethylene glycol 600-Nitroimidazole (Man-NIT), were developed to address the aggressive nature of hepatocellular carcinoma (HCC). Targeting GLUT1, overexpressed in cancer cells, the mannose shell enhances micelle uptake by HCCLM3 cells. Nitroimidazole reduces NADPH and glutathione levels, inducing reactive ROS and apoptosis. In vivo studies demonstrated significant tumor inhibition, marking Man-NIT as a promising nanodrug for HCC (Fig. 4 (b)) [45].

Researchers developed iBody, an N-(2-hydroxypropyl) methacrylamide-based copolymer combination with a CAIX inhibitor for specific targeting. The iBody has a strong affinity for CAIX, making it useful in biochemistry. Flow cytometry and confocal imaging can isolate CAIX from cell lysates and visualize it. These findings suggest that the iBody can target CAIX in vitro and potentially adapt for in vivo use [74]. A novel nanophotosensitizer, PS-02, was developed for targeting CAIX on tumor cells. PS-02 NPs exhibit a local "ET-cage effect," essential for regulating electron transfer (ET) processes. This regulation ultimately enhances the efficacy of Type I, PDT under hypoxic conditions, as demonstrated in mouse models, offering a new pathway for effective PDT [75]. Yu Yi et al. designed glucose-installed nanocarriers for targeted siRNA delivery to cancer stem cells (CSCs) via GLUT1. The assembly process involves glucose-modified polymers and gold NPs. These glucose-targeted NPs (Glu-NPs) achieve improved cellular uptake and gene silencing, effectively suppressing tumor growth in breast cancer models (Fig. 4(c)) [76].

A tumor-targeting lipid system has been engineered to deliver CRISPR/Cas9, targeting HIF-1α—a crucial factor in metastasis. This system coencapsulates Cas9, HIF-1α-targeting sgRNA, and paclitaxel (PTX) in liposomes modified with R8-dGR peptides, enhancing targeting and penetration. This strategy significantly inhibits tumor growth and metastasis, improving CRISPR/Cas9 applications for antimetastatic therapies (Fig. 4 (d)) [77].

Researchers developed sulfonyl-γ-AApeptides to disrupt the interaction between HIF-1α and p300 by mimicking HIF-1α′s helical domain. These peptides bind p300 with high affinity, inhibiting the hypoxia-inducible signaling pathway and selectively targeting hypoxia-related genes. The peptides, featuring both chiral and achiral sulfonyl side chains, effectively mimic protein helices involved in protein-protein interactions (PPIs). They are cell-permeable, stable, and exhibit favorable pharmacokinetics, suggesting their potential as a versatile strategy for modulating various PPIs [78].

### 4.4 Future applications and perspectives of NPs in hypoxic conditions

Recent advancements in nanotechnology emphasize the development of multifunctional nanoplatforms that integrate various treatment modalities, such as PDT, chemotherapy, and radiosensitizers [79,80]. These platforms support a holistic treatment approach that aligns with personalized medicine strategies. Specifically, advancements in targeting receptors like CAIX, GLUT1, and HIF within hypoxic tumor microenvironments demonstrate a shift toward precision medicine, enabling more effective targeted drug delivery systems. [81,82]. Research highlights the imperative need to address challenges in biocompatibility and potential systemic toxicity to facilitate successful clinical translation [83]. Monitoring long-term nanoparticle accumulation and their interactions with non-target tissues is crucial. Additionally, ensuring the scalability of nanoparticle production is a significant hurdle, necessitating solutions for batch-to-batch variability and economical mass production techniques to achieve market readiness [84,85]. Emerging studies suggest that theranostics could significantly improve personalized treatment plans by integrating diagnostic and therapeutic functions, thus enabling patient-specific interventions [86,87]. The potential synergies between hypoxia-targeting NPs and advanced genetic technologies, such as RNA therapies and CRISPR, may revolutionize precision oncology by offering customized and effective treatment protocols [88,89]. The ongoing exploration and development of hypoxia-targeting NPs are reshaping therapeutic landscapes by enhancing treatment specificity and efficacy. Bridging current knowledge gaps and leveraging existing opportunities could elevate cancer care and extend advancements to other medical fields, promising a future of improved and personalized healthcare solutions. However, challenges remain. Controlling the precise release and distribution of oxygen within TMEs could lead to treatment efficacy. The development of smart NPs capable of dynamically responding to oxidative environments is essential for real-time oxygen delivery adjustments. Combining oxygen-enhancing NPs with therapies like photothermal therapy might achieve synergistic effects, enhancing therapeutic outcomes [90,91].

Further understanding of the interactions between hypoxia-activated drugs and various tumor types is needed to optimize therapeutic windows and minimize potential side effects. Addressing the potential for resistance development due to prolonged hypoxia-targeted drug use remains crucial. Expanding the repertoire of hypoxia-activated compounds could provide more treatment alternatives and inform customized regimens. Additionally, deeper insights into the diversity of hypoxia-activated receptors across different cancer types are required to refine targeting strategies.

Evaluating the long-term efficacy and potential adaptive resistance to receptor-targeted NPs is an area necessitating further research [92,93]. Developing NPs capable of targeting multiple receptors concurrently could improve treatment success rates by addressing tumor heterogeneity. Continuous innovation of NPs to adapt within the dynamic TME will be pivotal in maintaining treatment efficacy over time.

## 5 Acidic pH targeting

The acidic microenvironment within cancer cells is a prominent characteristic that profoundly affects tumor development and progression. This acidic environment is a result of the altered metabolic state known as the Warburg effect, where cancer cells favor increased glucose uptake and anaerobic glycolysis, leading to the accumulation of lactic acid [94]. Clinicopathological evidence suggests that various transporters and pumps, including the Na+/H+ exchanger, H + -lactate co-transporter, and proton pump, contribute to H+ secretion and may be associated with tumor metastasis. A low oxygen level doesn't affect the acidic pH outside of cells; instead, it activates lysosomal enzymes that work best in an acidic range and increases the expression of pro-metastatic factor genes through an intracellular signaling cascade. Additional acidity comes from the pentose phosphate pathway CO2, showing the TME's hypoxia-extracellular acidification relationship [95].

To address these challenges, NPs can be engineered to be pH-responsive, allowing for targeted drug delivery and improved therapeutic efficacy while minimizing side effects on healthy cells [23]. Furthermore, strategies can also help restore a more physiological pH, thereby inhibiting tumor progression and enhancing the effectiveness of other therapeutic interventions [96]. Table 2 summarizes the NPs that are applied in targeting at acidic pH.

**Table 2:** Exploiting and Overcoming the TME by applying NPs in Acidic pH Targeting.

Strategies based on NPs	Techniques based on NPs	NPs
pH-Responsive Drug Release	Swell/release drugs in acidic tumor microenvironments	Polymeric NPs [97]
	Controlled release of therapeutics at tumor pH levels	Silica NPs [98]
	Release contents in acidic environments of tumors	Liposomes [99]
	pH-sensitive linkers release drugs in tumor sites	Gold NPs [100]
	Change in charge and size at different pH levels	Dendrimers [101]
	Degradation in acidic environments, releasing drugs	Chitosan NPs [102]
	Dissolve in acidic environments, releasing payloads	Lipid‐Coated Polyacrylic Acid/Calcium Phosphate NPs [103]
	pH-dependent release and targeted delivery using Gene delivery and drug delivery magnets	Magnetic NPs [104]
	Altered solubility and drug release at tumor pH	Poly(ethylene glycol) (PEG) NPs [105]
	Release drugs in response to pH changes	Micelles [106]
pH-Neutralization NPs	Reacts with acids to neutralize pH	carbonate NPs [107]
	Raised the extracellular pH (pHe) in tumor tissues	Sodium bicarbonate [108].

### 5.1 pH-responsive drug release

NPs can be designed to react to the tumor microenvironment's acidic pH. They may be made to release therapeutic compounds only when the environment becomes acidic, guaranteeing that cancer cells receive the targeted medication delivery. This method minimizes off-target effects while optimizing medication concentration at the tumor site [12,109]. Pancreatic ductal adenocarcinoma (PDAC) presents significant challenges for effective anticancer drug delivery due to its dense TME and inherent drug resistance [110]. Rosa Maria Iacobazzidet al. developed a microfluidic polymeric micelle (Gem@TpHResMic) drug delivery system for Gemcitabine (Gem) that is pH-responsive and uPAR-targeted. It produced micelles that had high drug-encapsulation efficiency, colloidal stability, and a restricted size distribution. To target Gemcitabine delivery in acidic TME settings, the micelles were designed to release it. They specifically targeted uPAR-expressing tumor cells, higher to free Gemcitabine in anticancer efficacy and cellular internalization. Gem@TpHResMic induced DNA damage and apoptosis in 2D and 3D pancreatic cancer models. In a co-culture model with cancer-associated fibroblasts (CAF), the micelles overcame TME-dependent drug resistance, boosting Gemcitabine's anticancer potential [111]. Jie Chang et al. developed mesoporous silica NPs (MSN-COOH) loaded with DOX, modified to form DOX@MSN-PEI-AA(DMPA) by adding polyethyleneimine (PEI) and anisamide (AA). This formulation facilitates tumor-specific entry via AA-mediated receptor endocytosis. In the acidic environment of lysosomes, PEI protonation enables DOX release into the cytoplasm. Hemolytic, in vitro, and in vivo studies confirmed DMPA's safety and precise targeting efficacy for breast cancer, highlighting its potential in tumor therapy [112].

**Fig. 5:**
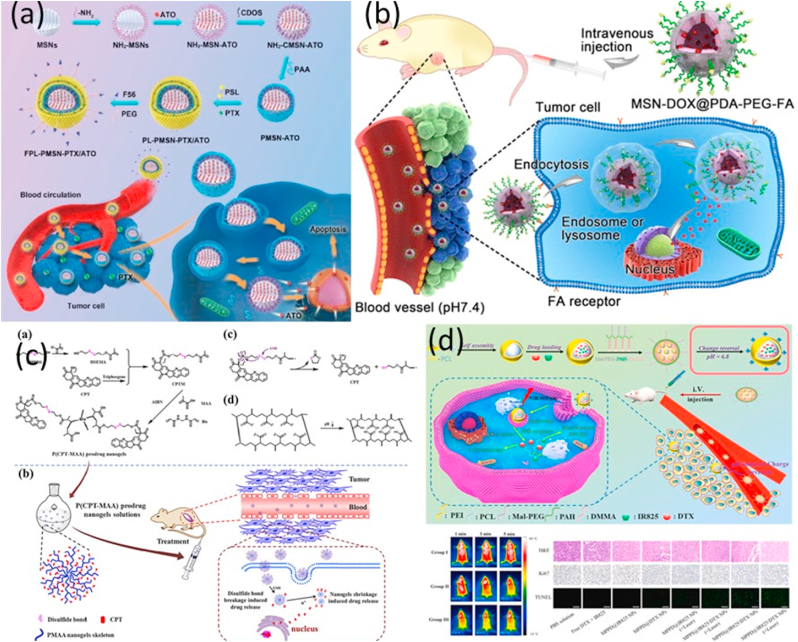
(a) Lipid/PAA-coated mesoporous silica NPs are utilized for the dual-pH-responsive delivery of arsenic trioxide/paclitaxel against breast cancer cells copyright [113] copyright (2021) nature (b) pH-Sensitive Delivery Vehicle Based on Folic Acid-Conjugated Polydopamine-Modified Mesoporous Silica NPs for Targeted Cancer Therapy [114]. Copyright (2017) American Chemical Society (c) Camptothecin prodrug nanogels that respond to both redox and pH provide "on-demand" drug delivery [115]. Copyright (2019) Elsevier (d) pH-responsive polymeric NPs loaded with IR825 and DTX exhibit synergistic chemo-photothermal cancer therapy with charge-reversal property [117]. Copyright (2022) Elsevier.

Bing-bing Zhang and colleagues designed mesoporous silica NPs (MSNs) coated with polyacrylic acid (PAA) and pH-sensitive lipid (PSL) to synergistically transport and release PTX and arsenic trioxide (ATO) (PL-PMSN-PTX/ATO) Fig. 5 (a) [113]. Cheng et al. created MSNs@PDA-PEG-FA, which was loaded with DOX, as a model drug for treating cervical cancer. They did this by attaching poly(ethylene glycol)-folic acid (PEG-FA) to mesoporous silica NPs (MSNs) that had been modified with polydopamine (PDA). In vitro drug release investigations indicated pH-dependent and sustained drug release characteristics that could boost anticancer effects and reduce normal cell harm from the tumor's acidic milieu. The in vitro cellular uptake and targeting assay showed that MSNs@PDA–PEG–FA had great targeting efficiency Fig. 5 (b) [114]. Camptothecin (CPT) was delivered using a new TME-responsive P(CPT-MAA) prodrug nanogel with reduced side effects. CPT was covalently integrated into distillation-precipitation polymerized nanogels utilizing a redox-responsive disulfide linker. The nanogels' spherical shape, consistent size, and dual pH/redox responsiveness allowed "on-demand" CPT release in the TME. The P(CPT-MAA) prodrug nanogels showed improved anticancer activity in vitro and in vivo without adverse effects, suggesting they could be a feasible chemotherapeutic agent delivery strategy Fig. 5 (c) [115].

IR825 is a NIR cyanine dye known for its distinct NIR absorbance and excellent thermal conversion performance, although it suffers from poor stability and limited therapeutic efficacy. Nanosystems which distribute NIR dyes have been designed to improve the management of tumors [116]. Xiaowei Wang et al. developed an intelligent polymer-based drug delivery system (MPPD/PEI-PCL) DTX and IR825 delivery via pH-responsive charge-reversal. In the acidic TME, these NPs increase cellular uptake and drug release. They demonstrated synergistic chemo-photothermal effects with NIR-triggered release, significantly improving antitumor efficiency in a 4T1 model. The formulation is biocompatible, offering targeted therapy for solid tumors. Fig. 5 (d) [117]. Zenjanab et al. developed a potential PTT combination therapy using niosomes (NIOs) carrying PTX and spherical gold NPs (AuNPs) coated with citrate, chitosan (Au-CS), or polyamidoamine (Au-PAMAM). Au-CS and Au-PAMAM coatings on NIOs increased PTX release due to their amino groups. The combined effects of chemotherapy (PTX) and photothermal therapy (AuNPs) increased cytotoxicity and death in breast cancer cells, demonstrating the promise of this technique for cancer treatment [12].

### 5.2 pH-neutralization NPs

pH-neutralization NPs are designed to counteract the acidic TME by releasing basic substances that neutralize excess hydrogen ions (H^+^). This neutralization can help to restore a more physiological pH, thereby inhibiting tumor progression and enhancing the effectiveness of other therapeutic agents. By neutralizing the acidic TME, these NPs can inhibit tumor cell proliferation and reduce metastatic potential [106,118]. In a study by Sandra F. Lam et al. RFP-expressing MDA-MB-231 were cultivated in fibrin-surrounded treatment and control chambers of a bifurcated microfluidic device by Sandra F. Lam et al. Control chamber got cell culture media, whereas the treatment chamber received acid-neutralizing CaCO3 NPs. Nano CaCO3 effectively corrected pH and dramatically inhibited tumor cell proliferation compared to the untreated control (p < 0.05). NanoCaCO3 incubated with fibroblasts inhibited MDA-MB-231 cancer cell proliferation and migration without killing fibroblasts. Nano CaCO3 treatment prolonged cancer cell intracellular pH decline, suggesting acidic TME metabolic reprogramming. Nano CaCO3's pH neutralization inhibited tumor growth and invasion while protecting stromal cells [119]. The suppressive tumor immune microenvironment (TIME) suppresses antitumor immune cells and allows tumor antigen mutation or deletion, limiting cancer immunotherapy efficacy. Weakly alkaline layered double hydroxide NPs (LDH NPs) neutralized excess acid and blocked tumor cell autophagy, improving neoadjuvant cancer immunotherapy. LDH NPs injected peritumorally neutralized acid in the TIME, inhibited tumor cell lysosome-mediated autophagy, and boosted antitumor tumor-associated macrophages and T cells. LDH NPs collected tissue-released tumor antigens, suppressing melanoma and colon tumor development in vivo. LDH NPs, as immunomodulators and adjuvants, stimulate and increase the host's innate and adaptive immune systems, presenting a promising basis for solid tumor immunotherapy by modifying the TIME and boosting immunological responses [120]. In another investigation, Rahman et al. used sodium bicarbonate (NaHCO3) orally and showed that it improved the antitumor effect of anti-PD-L1 antibody treatment. NaHCO3 raised the extracellular pH (pHe) in tumor tissues in vivo, which was followed by an increase in T cell infiltration, T cell activation, and upregulation of anti-tumor cytokines like IFN-γ, IL2, and IL12p40 in the tumor tissues. These modifications were further improved when NaHCO3 was administered in combination with anti-PD-L1 therapy, indicating a synergistic effect. It's interesting to note that the acidic extracellular conditions significantly increased PD-L1 expression in vitro, suggesting that the pH-normalizing effect of NaHCO3 can potentially bypass this immunosuppressive mechanism [108].

### 5.3 Future applications and perspectives of NPs in acidic pH conditions

The innovation in NPs that integrate both therapeutic and diagnostic capabilities—termed "theranostics"—is gaining significant momentum. These dual-function systems enable simultaneous tracking of drug delivery and therapeutic outcomes, thereby enhancing treatment personalization and efficacy [121]. Furthermore, the design of nanocarriers, like micelles and dendrimers, to be responsive to the TME acidic pH has increased their selectivity and reduced side effects, marking a noteworthy trend towards "smart" material applications in oncology. This strategic focus aligns with the broader ambition of precision medicine, where tailoring nanoparticle therapies to individual patient profiles becomes increasingly achievable. Advances in genomics and biomarker identification are pivotal in this transition, ensuring that therapies are suited to the most responsive candidates [23,122]. However, the intrinsic heterogeneity of the TME across different cancer types and stages challenges the uniform efficacy of pH-targeted therapies. The dynamic nature of the TME, with its fluctuating pH and hypoxia levels, complicates the sustained action of NPs crafted through fixed designs [123,124]. Moreover, the transition of NPs from lab-scale research to clinical applications often encounters obstacles due to scalability and toxicity concerns, alongside significant regulatory hurdles.

In response to these challenges, researchers are exploring NPs with multiple stimuli-responsive capabilities—including pH, temperature, and enzymatic activity—to better adapt to the TME's heterogeneity [125]. Sustained progress in nanoparticle synthesis and safety assessment is critical, with current efforts directed towards developing standardized frameworks to predict long-term toxicities. Additionally, further exploration of nanoparticle interactions within the TME is essential to inform new design principles for future therapeutic systems [126,127]. While the potential for innovation in targeting acidic pH conditions via nanoparticle therapies is vast, overcoming existing limitations will require concerted research initiatives that integrate molecular biology, materials science, and clinical expertise. Such collaborations are vital to fully capitalize on and translate these innovations into clinical success.

## 6 Immune modulation in the tumor microenvironment using NPs

TME presents a significant challenge for effective cancer immunotherapy due to its highly immunosuppressive nature. Nanoparticle platforms offer versatile solutions to modulate the immune response within the TME and enhance the efficacy of immunotherapeutic interventions.

NPs have emerged as a powerful tool in modulating the TME for enhanced cancer immunotherapy. These engineered particles can target the TME effectively, offering strategies like reprogramming immune cells and promoting immune responses [128]. By delivering agents directly to the TME, NPs can suppress elements like fibroblasts that inhibit effective immune responses, thus facilitating an anti-tumor immune environment [129]. Moreover, calcium carbonate NPs are noted for modulating the acidic environment of tumors, which can otherwise impede immune cell function [130]. Similarly, metallic NPs have demonstrated the capacity to adjust cytokine profiles within the tumor setting, thus enhancing immune-mediated attack on cancer cells [131]. These strategies highlight the potential of NPs not just in delivering drugs but also in re-engineering the TME to boost the body's natural immune response against tumors. The ongoing advancement in nanoparticle technology continues to open new pathways for effective cancer treatments. Table 3 summarizes the different NPs to immune modulation.

**Table 3:** Exploiting and Overcoming the TME by applying Nanoparticles to Immune Modulation.

Strategies based on nanoparticles	Techniques based on NPs	NPs
Targeting Immunosuppressive Macrophages by NPs	Encapsulation of immune-modulating agents to shift macrophage phenotype	Liposomes [132]
	Modulation of Macrophages M1/M2 Polarization	Polymeric NPs [133]
	Immune Modulatio	Lipid NPs [134]
	induced macrophage and inhibited cancer growth	Gold NPs [135]
	RNA interference to silence immunosuppressive genes	Mesoporous silica NPs [136]
Nanoparticle Activation of Tumor Immunostimulatory Cells	Promote T Cell-Mediated Immunotherapy	Dendrimers [137]

### 6.1 Targeting immunosuppressive macrophages by nanoparticles

**Fig. 6:**
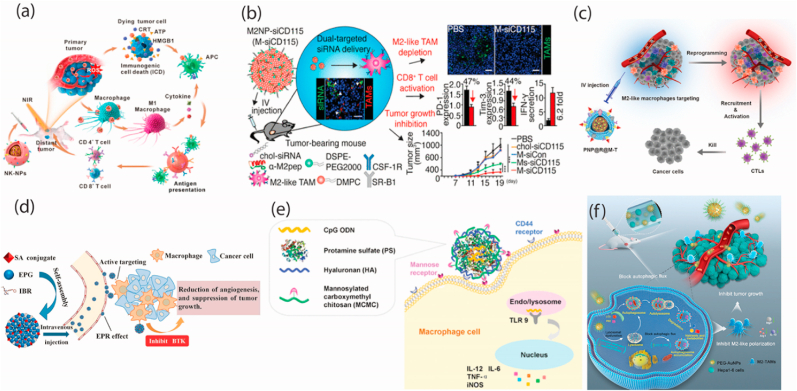
(a) Natural Killer Cell Membrane-Coated Nanoparticles are used in cell-membrane immunotherapy to stop the growth of both primary and metastatic cancer [141]. Copyright (2018) American Chemical Society (b) Novel molecularly targeted immunotherapy for melanoma using dual-targeting nanoparticles to deliver small interfering RNA to macrophages that are linked to tumors [146] copyright (2017) American Chemical Society (c) Development nanoparticles with Toll-like Receptor Agonists as a precise way to use immunotherapy to change the way tumor-associated macrophages work [147]. Copyright (2017) American Chemical Society (d) Targeted delivery of ibrutinib to tumor-associated macrophages by sialic acid-stearic acid conjugate modified nanocomplexes for cancer immunotherapy [148]. Copyright (2019) Elsevier (e) A Dual Macrophage Targeting Nanovector for Delivery of Oligodeoxynucleotides To Overcome Cancer-Associated Immunosuppression [149]. Copyright (2017) American Chemical Society (f) A Dual Macrophage Targeting Nanovector for Delivering Oligodeoxynucleotides to Overcome Immunosuppression Linked to Cancer [150] copyright (2022) Elsevier.

Tumor-associated macrophages (TAMs) are the dominant myeloid population, exhibiting a spectrum from immunosupportive M1-like to immunosuppressive M2-like phenotypes, the latter often prevailing in solid tumors due to hypoxic conditions [138]. The TME also harbors other immunosuppressive cells, such as regulatory T cells (Tregs) and myeloid-derived suppressor cells (MDSCs), which collectively create an environment that protects the tumor from immune attack, suppressing anti-tumor immunity. Modulating this complex, immunosuppressive TME is a critical challenge to improve cancer treatment efficacy [139]. Xinlong Zhang et al. have developed a lipid-coated calcium phosphonate NPs (CaP/miR@pMNPs) platform loaded with microRNA payloads. A pH-responsive material conjugates the NPs with mannose and shields them. In the acidic TME, the shielding is shed, exposing the mannose to facilitate selective internalization by immunosuppressive TAMs. This allows the NPs to reactivate and reprogram the macrophage functions, reversing the immunosuppressive state and inhibiting tumor growth in preclinical studies [140]. Deng et al. developed a cell membrane immunotherapy strategy using natural killer (NK) cell membrane-cloaked NPs loaded with a photosensitizer (NK-NPs). The NK-NPs targeted tumors, induced M1 macrophage polarization, and triggered photodynamic therapy-mediated cancer cell death, collectively enhancing antitumor immunity. This approach effectively eliminated primary tumors and inhibited distant tumors, showcasing the versatility of this cell-membrane-based immunotherapeutic strategy for comprehensive cancer treatment Fig. 6 (a) [141].

The eliminated NK cell membrane was extruded and placed on polymeric nanoparticles that contained photosensitizer TCPP. Through the NK cell membrane, the NK-NPs were able to initiate pro-inflammatory M1-macrophage polarization in the tumor, which resulted in cell-membrane immunotherapy. By inducing PDT-induced immunogenic cell death (ICD), NK-NPs can enhance the therapeutic efficacy of NK cell membranes by inducing dying tumor cells to release DAMPs (ATP secretion, CRT exposure, and HMGB1 release). Specifically, immunogenic PDT enhanced NK cell-membrane immunotherapy, which significantly promoted the infiltration of effector T cells (CD4^+^ and CD8^+^ T cells) in tumors for incredibly efficient tumor and abscopal tumor suppression [141].The anti-angiogenic therapies can normalize tumor vasculature, improving perfusion and reducing hypoxia to modulate the immunosuppressive TME [142]. For instance, low-dose anti-VEGFR2 antibody homogenized functional tumor vessels, relieving hypoxia and reprogramming immunosuppressive M2-like TAM towards an M1-like, pro-inflammatory state. This facilitated increased infiltration of CD4^+^ and CD8^+^ T cells, harnessing the immune system to overcome the hostile TME. By integrating vascular normalization and immunomodulatory mechanisms, this approach represents a promising strategy to enhance cancer immunotherapy efficacy [143,144]. Liu et al. developed a VEGFR-targeted drug delivery system utilizing a DSPE-PEG-NH2 linked, antibody-modified docetaxel-loaded lipid carrier. In a murine B16 tumor model, this system showed superior tolerance, antitumor activity, and cytotoxicity than existing treatments. It improved drug accumulation in tumors and vasculature, highlighting its potential as a promising cancer therapy [145].

Exposure to LPS and IFN-γ results in macrophage polarization in an M1 phenotype, which secretes IL-12 and inhibits cancer progression. In contrast, IL-4 and IL-13 induce M2 macrophages that secrete IL-10 and promote tumor growth [9]. M2-like TAM dual-targeting NPs (M2NPs) were designed by Yuan Qian et al. combining α-peptide and M2pep to improve targeting efficiency Anti-CSF-1R siRNA-loaded NPs remove M2-like TAMs from melanoma tumors, as shown by enhanced affinity and efficacy in tumor-bearing animals. With M2NPs, M2-like TAMs decreased 52 %, tumor size reduced 87 %, and survival increased. This method reduced immunosuppressive IL-10 and TGF-β, while increased IL-12, IFN-γ, and CD8^+^ T cell infiltration by 2.9-fold. Furthermore, it decreased exhaustion markers PD-1 and Tim-3 on CD8^+^ T cells and increased IFN-γ release by 6.2-fold, restoring T cell function Fig. 6 (b) [146]. Yun Zhang et al. developed M2-like macrophage-targeting NPs (PNP@R@M-T) that preferentially deliver drugs to M2-, M1-, and dendritic cells in 24 h in vivo. NPs altered 51 % of M2-like macrophages, decreased tumor growth by 82 %, and prolonged survival, making macrophage-mediated cancer immunotherapy successful Fig. 6 (c) [147]. Ibrutinib (IBR), a Bruton's tyrosine kinase inhibitor, shows promise for targeting TAMs involved in tumor progression and immunosuppression. However, its rapid clearance limits its effectiveness. To address this, researchers designed sialic acid–stearic acid nanocomplexes (SA/IBR/EPG) to encapsulate IBR, enhancing delivery and uptake by TAMs through SA-mediated targeting. These nanocomplexes demonstrated high IBR loading, prolonged circulation, and effective tumor delivery. In vitro and in vivo studies confirmed their preferential accumulation in TAMs, inhibiting angiogenesis and cytokine release, ultimately suppressing tumor growth without adverse effects. SA-decorated IBR nanocomplexes offer a promising cancer immunotherapy strategy Fig. 6 (d) [148]. A dual-targeting vector incorporating mannosylated carboxymethyl chitosan (MCMC)/hyaluronan (HA) and protamine sulfate delivered CpG oligodeoxynucleotides (ODN) to macrophages. This system enhanced immune activity by boosting proinflammatory cytokines and activating M1. In tumor cells (MCF-7), it increased NF-κB, PIK3R3, and p-Akt, while upregulating Fas/FasL, inducing apoptosis and raising caspase expressions. This approach has shown promise for overcoming cancer-associated immunosuppression Fig. 6 (e) [149]. Antitumor immunotherapy by suppressing TAMs M2 polarization with polyethylene glycol-conjugated gold NPs was investigated by Siyue Zhang et al. In vitro and in vivo, these NPs inhibited M2 polarization, boosting antitumor immunity and lowering mouse tumor development. PEG-AuNPs caused lysosome alkalization and membrane permeabilization in TAMs, inhibiting autophagic flux as shown by the mRFP-GFP-LC3 assay and autophagy-related protein analysis (LC3, beclin1, P62). This intervention reduced M2 polarization, revealing a mechanism where PEG-AuNPs induce antitumor effects through lysosome dysfunction and autophagy inhibition Fig. 6 (f) [150]. The IL@H-PP nanomedicine, using PEG-pHLIP-modified hollow copper sulfide nanoparticles, targets acidic tumors for enhanced PTT. By releasing ISRIB to inhibit stress granules, it sensitizes cancer cells to heat, boosts immunogenic cell death, and repolarizes TAM, transforming the microenvironment for intensified antitumor and anti-metastasis effects [151].

### 6.2 Nanoparticle activation of tumor immunostimulatory cells

**Fig. 7:**
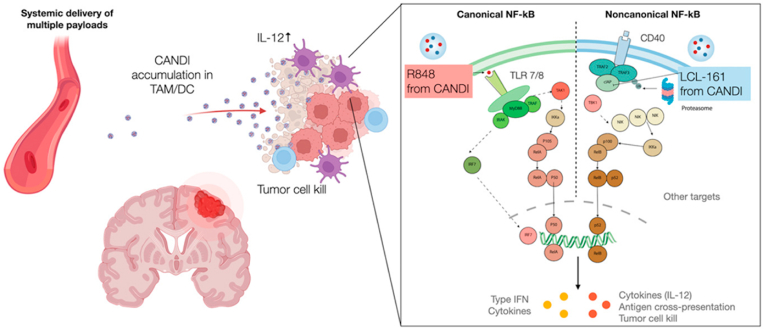
A strong immune response against glioblastoma in mice is triggered by a synthetic nanocarrier that blocks two immune pathways [152].

The TME's intricacy, tumor antigen hiding, and mutation promote immune escape while reducing immunostimulatory cells' anti-tumor effects [14]. Sophie Lug et al. have developed synthetic, cyclodextrin-adjuvant nanoconstructs (CANDI) that target tumor-associated myeloid cells in glioblastoma (GBM). To activate myeloid cells, these nanoconstructs contain a TLR7/8 agonist (R848) and a cIAP inhibitor (LCL-161). CANDI enters the GBM TME, aggregate, and are readily taken up by myeloid cells. The dual activation synergizes the payloads, enhancing immune activation compared to monotherapy, and increased T effector cells.The CANDI control GBM growth, showing efficacy both as a standalone treatment and in combination with anti-PD1 therapies, driving robust anti-tumor immunity (Fig. 7)[152].

Lorkowski et al. have developed a highly potent immunostimulatory nanoparticle (immuno-NP) that activates and expands antigen-presenting cells (APCs) within the tumor, inducing local interferon β secretion to recruit more APCs. The immuno-NP contains synergistic agonists for the stimulator of interferon genes (STING) and Toll-like receptor 4 (TLR4) pathways, enhancing IFNβ production and APC expansion. Systemic administration enables tumor deposition and predominant uptake by APCs, leading to significant expansion of dendritic cells and lymphocyte infiltration in a murine pancreatic cancer model [153]. To overcome the immunosuppressive TME, Covarrubias et al. have engineered an immunostimulatory NPs to reprogram dysfunctional APCs into activated, tumor-reactive APC subsets. Systemically delivered NPs efficiently targeted the APC-rich perivascular TME, predominantly being taken up by APCs. Loaded with a STING agonist, the NPs triggered robust interferon-β production, activating the reprogrammed APCs to stimulate cytotoxic T cells. Untargeted and integrin-targeted NP variants exhibited the most remarkable short- and long-term anti-tumor immune responses, highlighting the potential of this nanoparticle-mediated approach to reshape the TME and enhance cancer immunotherapies [154]. Cytotoxic T lymphocytes (CTLs) play a critical role in cancer immunotherapy, capable of directly killing tumor cells and secreting anti-tumor cytokines. Hyunjoon Kim et al. have developed novel TLR7/8 bi-specific agonist-loaded PLGA nanoparticles to potently activate antigen-presenting dendritic cells (DCs). These NPs migrated to draining lymph nodes, triggering DC activation and expansion, which led to enhanced antigen-specific CD8^+^ T cell responses. In preclinical tumor models, this approach demonstrated significant prophylactic and therapeutic efficacy, highlighting the promise of leveraging targeted DC activation to bolster cytotoxic T cell-mediated anti-tumor immunity [155]. Poly(lactic-co-glycolic) acid (PLGA) NPs show promise in drug delivery, particularly as peptide nanovaccines. Study by Chen et al. explores the in vivo effects of an intravenous (IV) STEAP peptide-loaded PLGA-NP, focusing on CD8^+^ T cell-mediated tumor suppression. Using prostate cancer mouse models, treatments included mSTEAP peptide in adjuvant via subcutaneous (SC) injection, IV or SC nanovaccine, CD8b mAb with nanovaccine, free peptide, or empty NPs. Enhanced CD8^+^ immune responses correlated with tumor inhibition and prolonged survival post-nanovaccine. Liver and lung accumulation was noted after IV nanovaccine. These findings highlight PLGA-based nanovaccines as viable prostate cancer immunotherapies [156]. Artificial antigen-presenting cells (aAPCs), created by incubating immature dendritic cells with PLGA nanoparticles containing tumor peptides and maturing with lipopolysaccharide, enhanced CTL responses. These aAPCs induced stronger, sustained tumor antigen-specific CTLs compared to controls. Frozen CTLs from aAPCs could effectively target HLA-A2-positive cancer cells, indicating aAPCs' potential superiority over DCs in immunotherapy, warranting clinical evaluation [157]. Poly(lactic-co-glycolic acid) NPs delivering p53 plasmid DNA restore p53 function, inhibiting tumor growth. In vivo, p53NPs show significant tumor suppression and survival improvement in p53-null prostate cancer mouse models, especially with intratumoral over intravenous administration. Imaging confirms NP tumor localization, suggesting potential for NP-mediated gene therapy in cancer. Further enhancements could improve targeting efficiency and therapeutic outcomes [158]. The SUN-NPA nanosystem, with acid-responsive shape transformation, self-delivers Chlorin e6 and immunomodulators metformin and sunitinib, ensuring homogeneous drug distribution for effective photodynamic therapy (PDT). By alleviating tumor immunosuppression, SUN-NPA enhances PDT and activates robust antitumor immunity, significantly inhibiting tumor growth and metastasis, showcasing its strong oncotherapy potential [159]. A novel carrier-free nanodrug system self-assembles for precise drug delivery in breast cancer. It uses p-phthalaldehyde, dithiodipropionic acid, and an MMP-2 cleavable peptide for targeted drug release. This system spatially delivers metformin, DIP, and SN38, reversing tumor immunosuppression, and inhibiting metastasis with high precision in the tumor microenvironment [160]. The NLG919@Lip-pep1 liposome system intelligently targets breast cancer cells, using PD-1 pathway inhibitor AUNP-12 and IDO inhibitor NLG919. Triggered by tumor MMP-2, it enhances T cell activity, modulates the immunosuppressive microenvironment, and precisely delivers drugs, offering efficient, low-toxicity therapy for metastatic breast cancer through targeted immunotherapy [161]. The D-HCuS@HA nanoparticles, enhanced with losartan, improve PTT by targeting breast tumors and increasing photothermal agent accumulation. Upon NIR irradiation, they release Cu(DDTC)2, eliminating resistant tumor cells and inducing effective immunogenic cell death, while losartan remodels the tumor microenvironment, promoting T cell infiltration to combat metastasis [162].

### 6.3 Future applications, challenges and perspectives of NPs in immune modulation conditions

NPs represent a cutting-edge approach in modulating the immune landscape of the TME, offering innovative strategies to amplify the efficacy of cancer immunotherapies [163]. A notable trend in this realm is the development of NPs designed to alter macrophage polarization—from a tumor-promoting M2 phenotype to a tumor-fighting M1 phenotype. This transformation is pivotal in dismantling the immunosuppressive barriers present within the TME [164]. Polymer-based NPs and lipid-coated nanostructures, which are loaded with immune-modulating agents, have shown effectiveness in preclinical models [164,165]. Despite these promising preclinical outcomes, transitioning nanotherapies from the laboratory setting to clinical practice faces significant challenges, including variable efficacy, regulatory hurdles, and safety assurance in humans [166]. The inherent complexity of the immune system, coupled with the dynamic nature of the TME, complicates the prediction of immune modulation outcomes by NPs. Therefore, ongoing research is focused on thoroughly mapping these interactions. Moreover, tailored NPs can effectively deliver checkpoint inhibitors directly to the TME, thereby increasing their concentration at tumor sites while minimizing systemic side effects. Such targeted delivery systems are anticipated to boost the efficacy of therapies like PD-1 and CTLA-4 blockade [[166], [167], [168]]. Advanced designs now allow for the concurrent delivery of multiple therapeutic agents—including immunomodulators, cytokines, and chemotherapeutics within a single nanoparticle. This multifaceted approach leverages synergies to enhance cancer treatment, enabling simultaneous immune stimulation and direct tumor attack. The variability of the immune microenvironment between different patients presents an additional layer of complexity, especially in standardizing nanoparticle treatments [169]. Personalized approaches, potentially informed by genomic analysis, may be essential to effectively address these differences [[169], [170], [171]]. Furthermore, it is imperative to comprehensively understand the long-term impacts of NPs on immune function and overall health, necessitating the development of rigorous regulatory pathways to ensure safety. Looking to the future, there is potential in developing adaptive NPs capable of dynamically interacting with the immune environment, adjusting their therapeutic payload based on real-time feedback from the body and the tumor. The integration of biosensors to monitor immune responses can allow for the real-time optimization of NPs based on patient-specific responses, thus maximizing therapeutic efficacy. Concurrent advances in manufacturing technologies are paving the way for more efficient development and scaling of these sophisticated nanoparticle systems, facilitating their progression from bench to bedside. Collaborations among regulatory bodies, industry, and research institutions will be vital in overcoming translation barriers [170,172,173].

Ultimately, the strategic deployment of NPs in immune modulation not only holds the promise of revolutionizing cancer immunotherapy but also emphasizes the significance of personalized and adaptive therapeutic strategies in overcoming the inherent challenges presented by the tumor microenvironment.

## 7 Nanoparticle-mediated targeting of the CAFs

The TME is a complex ecosystem, with CAFs playing a critical role in mediating immunosuppression and promoting tumor progression. Fibroblasts play a crucial role in maintaining the structural and functional integrity of healthy tissues. They achieve this by depositing the extracellular matrix, which provides the necessary scaffolding, and by promoting tissue repair processes. By upregulating factors like α-smooth muscle actin (α-SMA), pro-angiogenic signals, and VEGF, CAFs create a physical and biochemical barrier that hampers effective penetration of therapeutic agents [174]. Nanoparticle strategies can either disrupt the CAF compartment or leverage CAFs to enhance therapeutic efficacy, remodeling the TME and overcoming this critical cancer-driving pathology. Targeting the myofibroblast subset within the CAF compartment is a promising strategy [174]. Myofibroblasts, driven by TGF-β1-mediated ROS signaling and α-SMA upregulation, exhibit pro-invasive and tumor-supportive functions. Notably, this myofibroblast differentiation can be suppressed by antioxidant compounds, offering a means to remodel the TME [175]. Alili et al. have reported promising findings on the use of nanoceria to regulate the CAF compartment. They demonstrated that nanoceria can selectively downregulate the expression of α-SMA, a key marker of the pro-tumorigenic myofibroblast phenotype. Additionally, these nanoparticles were able to inhibit the invasiveness of squamous cancer cells. Importantly, the nanoceria exhibited specificity towards the tumor cells, suggesting a safe and effective therapeutic approach to target the CAF-driven pathology [176]. Table 4 briefly lists NPs that are applied for CAFs modulation.

**Table 4:** Exploiting and Overcoming the TME by applying NPs to CAFs Modulation.

Strategies based on NPs	Techniques based on NPs	NPs
Drug delivery to CAF	Enhancing drug penetration in CAF	Lipid-based NPs [177]
Modulation of CAF Behavior	Altering CAF activation states (from pro-tumor to anti-tumor)	Polymeric NPs [178]
Inhibition of CAF-mediated Immunosuppression	Inducing apoptosis in CAFs	Gold NPs [179,180]
RNA Interference toTarget CAFs	Silencing genes associated with CAF activation	Mesoporous silica NPs [181,182]
Enhanced Drug Delivery via CAFs	Targeting drug delivery specifically to CAFs	Dendrimers [180,183]

**Fig. 8:**
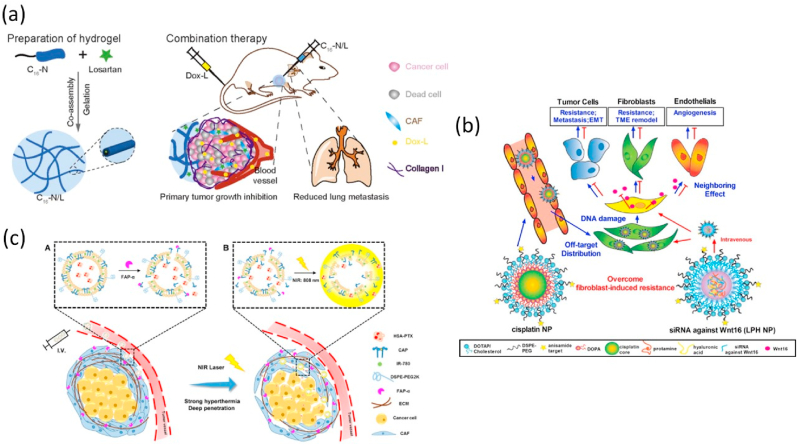
Nanoparticle-mediated targeting of the CAFs: (a) Regulating CAFs with losartan-loaded injectable peptide hydrogel to potentiate chemotherapy by inhibiting the growth and lung metastasis of triple-negative breast cancer. Copyright by (2017) Elsevier [184] (b) investigating the effects of NP damaged TAFs on neighboring cells and alteration of stromal structure after cisplatin treatment. Copyright by (2015) Elsevier [186] (c) Dual-responsive lipid-albumin nanoparticles target CAFs to improve drug perfusion for pancreatic tumor therapy. Copyright (2015) Elsevier [190].

Hu et al. rationally designed, screened, and evaluated an injectable peptide hydrogel as a local losartan depot. The target was CAF inhibition and chemotherapeutic enhancement. C16-GNNQQNYKD-OH (C16-N) outperformed the others in hydrogel formation and drug encapsulation after peptide derivative synthesis. After intratumoral injection, the C16-N hydrogel persisted in the tumor for over 9 days, suppressed CAFs and collagen formation, and increased PEGylated doxorubicin-loaded liposome effects on tumor growth and metastasis. This study provides key ideas for rationally designing injectable peptide hydrogels to modulate CAFs and improve chemotherapy (Fig. 8 a) [184].

In addition to disrupting CAFs, another approach involves directly targeting these cells with cytotoxic agents. By eliminating CAFs, this strategy can overcome their drug resistance-promoting functions, including the upregulation of proteins like Wnt16 and the creation of physical barriers that impede drug delivery [185]. Miao et al. showed that cisplatin-damaged CAFs upregulate Wnt16, which causes drug resistance. This was solved by creating liposome-protamine-hyaluronic acid nanoparticles with anti-Wnt16 siRNAs. These NPs boost cisplatin-loaded lipid-calcium-phosphate nanoparticle efficacy in stroma-rich bladder cancer. Even in advanced disease, the dual nanoparticle method significantly boosted antitumor responses. Wnt16 downregulation resensitized cancer cells to cisplatin, remodeled the TME and fibroblast populations, and inhibited angiogenesis. This novel strategy shows that targeting CAF-derived factors like Wnt16 can overcome drug resistance and improve cancer treatments. By blocking Wnt16 in injured CAFs, nanoparticle-based combination treatments can overcome cisplatin resistance and resensitize the TME (Fig. 8 b) [186]. Quercetin-loaded LCP NPs inhibited Wnt16 in CAFs, therefore the same team of researchers investigated their medicinal potential. Quercetin-loaded nanoparticles downregulated Wnt16 and synergistically inhibited bladder cancer in a stroma-rich bladder cancer model with cisplatin NPs. This unique method targets CAFs and resensitizes tumors to cancer therapy by altering the TME and increasing nanoparticle absorption [187]. Huang et al. loaded targeted plasmids encoding TNF-related sTRAIL onto lipid-coated protamine DNA nanoparticles. Desmoplastic bladder cancer xenografts received sTRAIL NPs. Specifically, sTRAIL caused apoptosis in malignant cell nests around CAFs. It also converted CAFs into quiescence, ablating the tumor and changing the TME to improve nanotherapeutic therapies [188]. NPs can target and change CAFs, suggesting a viable treatment for desmoplastic, stroma-rich solid tumors. Pancreatic ductal adenocarcinoma (PDAC) has a thick tumor stroma that prevents medication delivery, making treatment difficult [189]. Yu et al. designed a nanoparticle technology that responds to the CAF membrane biomarker FAP-α and NIR laser irradiation to tackle this difficulty.The NPs contained photothermal IR-780 and tiny albumin-bound paclitaxel (HSA-PTX) with strong tumor-penetrating ability. The FAP-α-responsive and thermosensitive liposome design (CAP-ITSL) focused chemotherapeutic administration to CAF-rich tumor stroma. The photothermal effect of NIR laser irradiation enlarged the tumor interstitial space, boosting HSA-PTX release deep inside the tumor [190].

Celastrol and betulinic acid-loaded micelles were co-encapsulated in liposomes (CL@BM) and modified with folic acid for targeting. NIH3T3 and 4T1 cells were co-cultured to test the nanocarrier's capacity to suppress CAF-induced drug resistance and migration. In a triple-negative breast cancer model, the F/CL@BM nanocarrier outperformed the drug formulations in biodistribution, safety, and anti-tumor and anti-metastatic activities [177].

### 7.1 Future applications, challenges and perspectives of NPs in CAF conditions

NPs can be engineered to target CAFs specifically, disrupting their signaling pathways and impairing their ability to remodel the extracellular matrix and promote tumor growth. [191,192]. Despite the promise of NPs in cancer therapy, their toxicity remains a significant concern. NPs can induce adverse immune responses or accumulate in organs, causing unforeseen side effects [193,194]. Maintaining NP stability in biological environments is crucial to prevent premature degradation or aggregation, which could lead to off-target effects and diminished therapeutic efficacy. Research should focus on integrating advanced targeting ligands or antibodies that specifically hone in on CAFs, reducing potential side effects on healthy tissues [195]. Multifunctional NPs, which can co-deliver chemotherapeutics and agents that inhibit CAF-activated signaling pathways, hold potential to yield synergistic therapeutic effects. By simultaneously targeting CAFs and cancer cells, these NPs could reduce tumor stroma density, enhance drug penetration, and consequently improve the overall efficacy of cancer treatments [196]. Future applications of NPs will likely focus on improving the selectivity and specificity of NPs towards CAFs. This includes using advanced targeting moieties such as ligands or antibodies that bind specifically to biomarkers commonly overexpressed on CAFs like FAP [197]. Leveraging data and insights from genomics and proteomics to customize NP therapies for individual patients may dramatically increase effectiveness and safety. Advancements in these areas could significantly enhance the clinical feasibility of NP-based technologies, potentially transforming the treatment landscape of cancer by effectively targeting CAF and other [198,199].

Enhancing the penetration and cellular uptake of these nanoparticles, particularly in tumors with dense stromal content like pancreatic and triple-negative breast cancers, remains an active area of research.

## 8 Modulating the tumor extracellular matrix with NPs

NPs offer innovative strategies to modulate the tumor ECM. These carriers can selectively target and degrade ECM components like collagen and hyaluronic acid, improving drug penetration and distribution within the tumor [200]. By remodeling the dense, desmoplastic tumor stroma, nanoparticles enhance the delivery and efficacy of co-administered cancer therapies, including chemotherapies and immunotherapies. A number of studies have developed artificial molecules that imitate the ECM's role in tumor growth and prevent metastasis, a leading cause of cancer death. Yata et al. examined when ECM depletion affects AAVP targeting in 2D monolayers and 3D tumor spheroid models using various tumor cell lines. AAVP diffusion, internalization, gene expression, and cytotoxicity increased with collagenase and hyaluronidase treatment of ECM collagen, hyaluronic acid, and fibronectin. The work shows that ECM depletion dramatically increases AAVP-mediated gene transfer, underlining the benefit of ECM modification in therapy. Results from 3D multicellular tumor spheroid (MCTS) models closely resemble in vivo settings, highlighting their clinical importance [201]. Yang et al. designed a collagenase-functionalized, biomimetic gold nanoplatform for PDAC therapy. This system integrates ECM degradation, tumor targeting, and NIR-triggered drug release for enhanced antitumor effects and diagnostics. Constructed by coating doxorubicin-loaded Au nanocages with PDAC cell membranes, followed by collagenase attachment, this nanoplatform targets tumors effectively, degrades ECM to enhance drug delivery, and under NIR, provides photothermal, photodynamic, and chemotherapy while enabling CT imaging for therapy monitoring [202]. In another study, a novel HA@PRB/COL NPs system was developed by Wang et al. to target hepatic fibrosis. By combining probucol and collagenase type I, this system effectively degraded ECM collagen, enhancing drug delivery to hepatic stellate cells (HSCs). Through CD44 receptor specificity, the NPs penetrated fibrotic liver, inhibiting HSC activation and autophagy. Results in a mouse model demonstrated efficient anti-fibrotic effects [203]. Hu et al. developed a laminin-mimicking peptide for an artificial ECM to inhibit tumor invasion because laminin is essential to neoplastic ECM. This mimic self-assembled into nanofibers when it bound to tumor cell laminin receptors. ECM-like NPs retained at neoplastic sites for 3 days, preventing metastasis in various solid tumor types. Cell-adhesive patterns imitating tumorous ECM were created using magnetic nanocarriers on agarose hydrogel under magnetic fields. This technique directed cell behavior, promoting osteoclast aggregation. Biomimetic nanoparticles demonstrate unique ECM integrity tools that affect adhesion and metastatic dynamics [204]. Table 5 provides an overview of NPs applied for ECM modulation.

**Table 5:** Exploiting and Overcoming the TME by applying Nanoparticles to ECM Modulation.

Strategies based on nanoparticles	Techniques based on NPs	NPs
ECM Component Degradation	Target degradation of ECM components like collagen to enhance drug penetration.	Albumin NPs, Biomimetic drug-loaded Au nanoplatform [202,205].
Targeting Pancreatic Tumor Stroma and ECM	NPs facilitate the crossing of dense ECM and stroma to deliver antitumor drugs or cells. Targeting via ligand-mediation (peptides, siRNAs) and stimuli-responsive properties (acidic/hypoxic).	Liposomes, Polymeric NPs, Inorganic NPs [206].
Detection of LOX Activity for ECM Analysis	Utilize gold NPs that aggregate and change color upon exposure to LOX, enabling a colorimetric assay for detection	Peptide-Functionalized Gold NPs [207].

### 8.1 Future applications, challenges and perspectives of NPs in ECM conditions

NPs present promising solutions for modulating the tumor ECM, yet several ECM-specific challenges remain to be addressed to fully realize their clinical potential. The tumor ECM is inherently heterogeneous, with its composition and structure varying significantly across different tumor types and even within individual tumors [208,209]. This heterogeneity complicates the universal application of nanoparticle strategies. To tackle this, future research must aim to map the diverse compositions of ECMs and develop adaptable nanoparticle platforms that can tailor their interventions to the specific characteristics of an ECM [210].

Targeting ECM components such as collagen and hyaluronic acid with precision is crucial, as off-target degradation could lead to adverse effects like inflammation or damage to nearby healthy tissues [211]. Therefore, it is essential to advance targeting mechanisms and integrate real-time monitoring of ECM-modifying actions to mitigate such risks.

The ECM's dynamic nature, characterized by continuous remodeling influenced by factors like tumor growth, metastasis, and therapeutic interventions, poses challenges for the sustained delivery and efficacy of NP therapies [212,213]. Innovations in creating smart nanoparticles that can adapt to these ECM changes in real time, potentially through stimulus-responsive components, will be fundamental in overcoming these barriers.

Furthermore, transitioning successful ECM-modulating nanoparticle strategies from preclinical models to clinical applications involves a series of hurdles. These include validating the efficacy of ECM-targeting in diverse patient populations and integrating nanoparticle-mediated ECM modulation seamlessly into existing treatment regimens [214,215]. Addressing these challenges will necessitate comprehensive clinical studies and innovative trial designs.

These ECM-focused challenges not only underscore the complexities involved in utilizing nanoparticles for tumor ECM modulation but also highlight the necessity.

## 9 Conclusion

In conclusion, the innovative application of NPs to modulate the intricate TME represents a transformative step in cancer therapy. By precisely targeting critical features such as hypoxia, acidity, and immune suppression, nanoparticles provide an integrated approach to enhancing treatment efficacy and overcoming therapeutic resistance. This paper underscores the paramount importance of addressing the multifaceted nature of the TME. The potential of nanotechnology to revolutionize cancer treatment strategies is underscored through its capacity to navigate these complexities. Furthermore, key gaps remain to be addressed in our understanding of the TME's dynamic interactions with nanoparticles. Future research should focus on elucidating these interactions and designing nanoparticles that can adapt to the evolving conditions within the TME. Personalized and context-specific therapies could emerge from such investigations, tailored to unique patient profiles and the heterogeneous nature of cancer environments. Embracing multidisciplinary collaboration and innovative research methodologies will be crucial in the continued exploration and refinement of nanoparticle-based interventions. Such endeavors hold the promise of not only advancing scientific knowledge but also ushering in a new era of targeted, impactful, and clinically relevant cancer therapies. This progress could significantly shift the paradigm of cancer treatment, moving towards more effective, personalized, and prognostically favorable outcomes for patients.

## CRediT authorship contribution statement

**Soroush Karimi:** Writing – original draft, Methodology, Investigation, Conceptualization. **Roksana Bakhshali:** Resources, Methodology, Investigation. **Soheil Bolandi:** Resources, Methodology, Investigation. **Zahra Zahed:** Investigation, Methodology, Resources. **Seyedeh Sahar Mojtaba Zadeh:** Visualization, Methodology, Investigation. **Masoumeh Kaveh Zenjanab:** Writing – original draft, Visualization, Methodology, Investigation, Conceptualization. **Rana Jahanban Esfahlan:** Writing – review & editing, Validation, Supervision, Project administration, Funding acquisition, Conceptualization.

## Availability of data and materials

Not applicable.

## Declaration of competing interest

The authors declare that they have no known competing financial interests or personal relationships that could have appeared to influence the work reported in this paper.

## Data Availability

No data was used for the research described in the article.
